# Mapping and Surgical Ablation of Focal Epicardial Left Ventricular Tachycardia

**DOI:** 10.1155/2011/471397

**Published:** 2011-09-13

**Authors:** Arif Elvan, Hauw T. Sie, Anand R. Ramdat Misier, Andre C. Linnenbank, Peter Paul H. M. Delnoy, Jacques M. T. de Bakker

**Affiliations:** ^1^Department of Cardiology, Isala Klinieken Zwolle, Groot Wezenland 20, 8011 JW Zwolle, The Netherlands; ^2^Department of Cardiothoracic Surgery, Isala Klinieken Zwolle, 8011 JW Zwolle, The Netherlands; ^3^Department of Experimental Cardiology, Academic Medical Centre, 1105 AZ Amsterdam, The Netherlands

## Abstract

We describe a technical challenge in a 17-year-old patient with incessant epicardial focal ventricular arrhythmia and diminished LV function. Failure of ablation at the earliest activated endocardial site during ectopy suggested an epicardial origin, which was supported by specific electrocardiographic criteria. Epicardial ablation was not possible due to the localization of the origin of the ventricular tachycardia adjacent to the phrenic nerve. Minimal invasive surgical multielectrode high-density epicardial mapping was performed to localize the arrhythmia focus. Epicardial surgical RF ablation resulted in the termination of ventricular ectopy. After 2 years, the patient is still free from arrhythmias.

## 1. Introduction

Despite the success of catheter ablation for treatment of idiopathic ventricular tachycardia (VT), occasional patients have been reported in whom VT could not be ablated from the right or left ventricular endocardium or from the aortic sinus of Valsalva. Although clinically underrecognized, idiopathic ventricular ectopy or VT may originate from the left ventricular epicardium. The mechanism is consistent with abnormal automaticity or triggered activity. In the absence of heart disease and if ventricular ectopy is infrequent with no documented VT, patients, especially if they are relatively asymptomatic, can be reassured, and no specific treatment is required. In patients with normal hearts and frequent monomorphic ventricular ectopy and VTs, catheter ablation should be considered. In general, these ventricular arrhythmias are difficult to treat with antiarrhythmic drugs. In the majority of patients, it is amenable to perform ablation by transvenous, retrograde arterial, or transpericardial approaches. The technique of percutaneous epicardial ablation was pioneered by Sosa et al. [1] for the ablation of VT in patients with Chagas disease. One of the limitations of epicardial ablation is the potential proximity of the left phrenic nerve [2–4]. 

This paper describes a technical challenge in a patient with epicardial focal ventricular ectopy. Epicardial ablation was not possible due to localization of the origin of the ventricular tachycardia adjacent to the phrenic nerve.

## 2. Case Report

**Figure 1:**
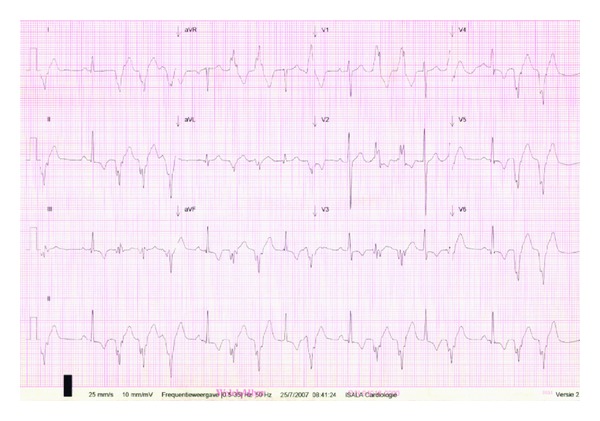
Twelve lead ECG showing ventricular bigeminy, doublet PVC's with an RBBB configuration with an RS interval of >100 msec in the precordial leads, and a pseudodelta wave configuration suggesting an epicardial origin of the ventricular ectopy.

This paper describes a 17-year-old female who developed fatigue and palpitations in 2005. She was referred to a local cardiologist for the evaluation of an irregular pulse, which was subsequently found to be caused by monomorphic premature ventricular complexes (PVCs) and nonsustained monomorphic ventricular tachycardias (VTs). The 12 lead ECG showed a PVC with an RBBB morphology (Figure 1). Echocardiography showed a dilated left atrium and a dilated left ventricle with decreased LV function. Preablation MRI images were of poor quality due to frequent PVCs and nonsustained VTs. The result of real-time cardiac MRI was decreased LV function (LVEF 40%) and the absence of late enhancement. This patient had a high burden of ectopic ventricular activity: 24 hours of ambulatory electrocardiogram recordings showed a maximal PVC count of 200 PVCs per minute and with a maximum of 2867 PVCs per hour. The maximal number of doublet PVCs was 600 per hour. Ventricular ectopy, bigeminy, and nonsustained VT's were monomorphic and persisted during the 24 hour period. The data suggested that the frequent occurrence of PVCs and nonsustained ventricular tachycardias were associated with inadequate cardiac output due to a functional bradycardia. This was the most likely explanation for her fatigue. 

The patient had undergone multiple antiarrhythmic drugs trials. Amiodarone, flecainide, quinidine, sotalol, metoprolol, and verapamil have been administered, all without sustained success. The patient had undergone 2 radiofrequency catheter ablation attempts. Because of her progressive complaints of fatigue and palpitations, she was referred for a third catheter ablation procedure to our hospital. A combined endocardial and epicardial mapping procedure was planned.

## 3. EP Study and RF Catheter Ablation

The patient was transferred to the cardiac catheterization laboratory, where a diagnostic quadripolar catheter (6Fr, Josephson, Bard) was placed at the right ventricular apex via the right femoral vein and a cooled tip mapping catheter (deflectable 8Fr, D curve, Biosense Webster) was placed in the LV via the right femoral artery. 5 000 units of heparin IV was given and together with 2 500 to 5 000 unit IV boluses to maintain an activated clotting time between 250 and 300 s. 12-lead ECG and intra-arterial pressure were continuously monitored. The mapping catheter was moved around the endocardial surface of the LV to detect the area with earliest local activation. The ablation catheter was, however, unstable at the site of earliest activation. Therefore, a transseptal puncture was performed to access the LV via the mitral valve, which resulted in a more stable position of the catheter. The site of earliest endocardial activation was localized at the antero-apico-lateral side of the left ventricle. Despite extensive mapping and radiofrequency energy applications, only temporary termination of VT was accomplished. This was a suggestive clue for considering an epicardial location of the origin of the arrhythmias.

## 4. Percutaneous Epicardial Mapping and Ablation

**Figure 2:**
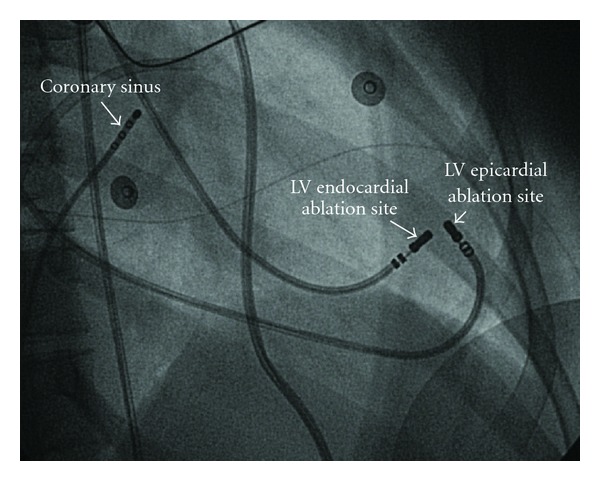
Fluoroscopic image showing the ablation catheters at the site of earliest activation endocardially and epicardially. The tip of the ablation catheters was located at the apicolateral side of the left ventricle. Ablation endocardially resulted in temporary elimination of the ectopy. Epicardially RF energy delivery was associated with phrenic nerve stimulation.

Following cleaning and draping of the patient, a fluoroscopically guided pericardial puncture was performed via the sub-xiphisternal approach. As the needle was advanced, small boluses of half-strength contrast were injected to identify when the pericardial space was entered. Then, using the Seldinger technique, a standard 8Fr sheath was exchanged over the guidewire. A cooled tip quadripolar ablation catheter (Biosense Webster) was inserted into the pericardial space and manipulated easily to areas corresponding to the endocardial sites at which ablation had previously failed (Figure 2). Pacing was performed to delineate the course of the left phrenic nerve. Epicardial RF energy delivery at the site with earliest activation caused diaphragm stimulation. This suggested a focus adjacent to the left phrenic nerve. Therefore, the procedure was terminated. An epicardial surgical procedure was planned 2 weeks after the failed percutaneous approach. 

## 5. Surgical Mapping and Ablation Procedure

**Figure 3:**
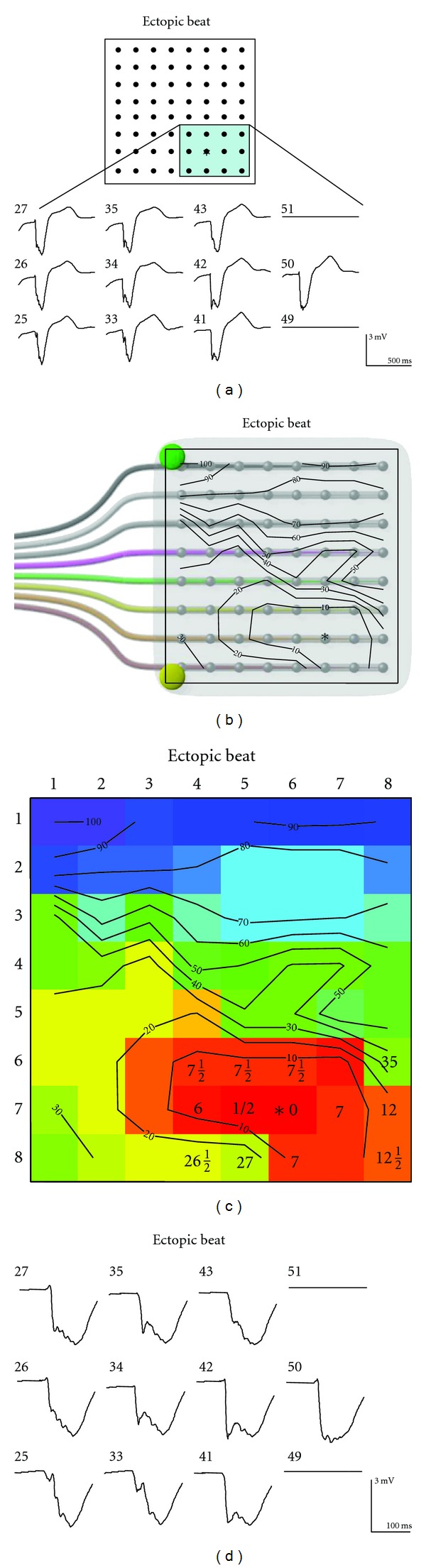
Multielectrode high density mapping was performed using a rectangular grid electrode harboring 64 electrode terminals arranged in an 8 × 8 matrix (interelectrode distance 5 mm). The electrode was positioned over the area where the origin of the tachycardia was expected. The earliest site of epicardial activation was located at the apicolateral side of the left ventricle. The numbers on the left side of the unipolar electrograms refer to the grid electrodes (a). The unipolar electrogram recorded at the earliest activated site was initially negative, suggesting that activation spread from this site. Isochronal maps depict the centrifugal activation pattern of the ectopic beats (b and c). Electrograms at sites adjacent to the earliest activated site were fractionated, suggesting discontinuous conduction (d). See text for details.

Following general anesthesia, a 5 cm incision at the fourth intercostal space in the anterior axillary line was made. Defibrillator pads were placed on the thoracic wall. The course of the phrenic nerve over the pericardium was identified, and the pericardium was opened anterior to the phrenic nerve. Multielectrode high density mapping was performed using a rectangular grid electrode harboring 64 electrode terminals arranged in an 8 × 8 matrix (interelectrode distance 5 mm, Figures 3(a) and 3(b). The electrode was positioned over the area where the origin of the tachycardia was expected. The site of earliest epicardial activation was located at the apicolateral side of the left ventricle (Figures 3(a) and 3(b)). The unipolar electrogram recorded at the earliest activated site was initially negative, indicative for an origin of activation at that site (electrogram in Figure 3(a)). The activation map revealed centrifugal spread of activation from the earliest activated site (Figure 3(c)). Electrograms at sites adjacent to the earliest activated site were fractionated, suggesting discontinuous conduction (Figure 3(d)). After epicardial cooled tip RF application, ventricular ectopy was neither spontaneously present nor inducible by programmed stimulation. Six months after ablation, the patient is still free from ventricular arrhythmias. 

**Figure 4:**
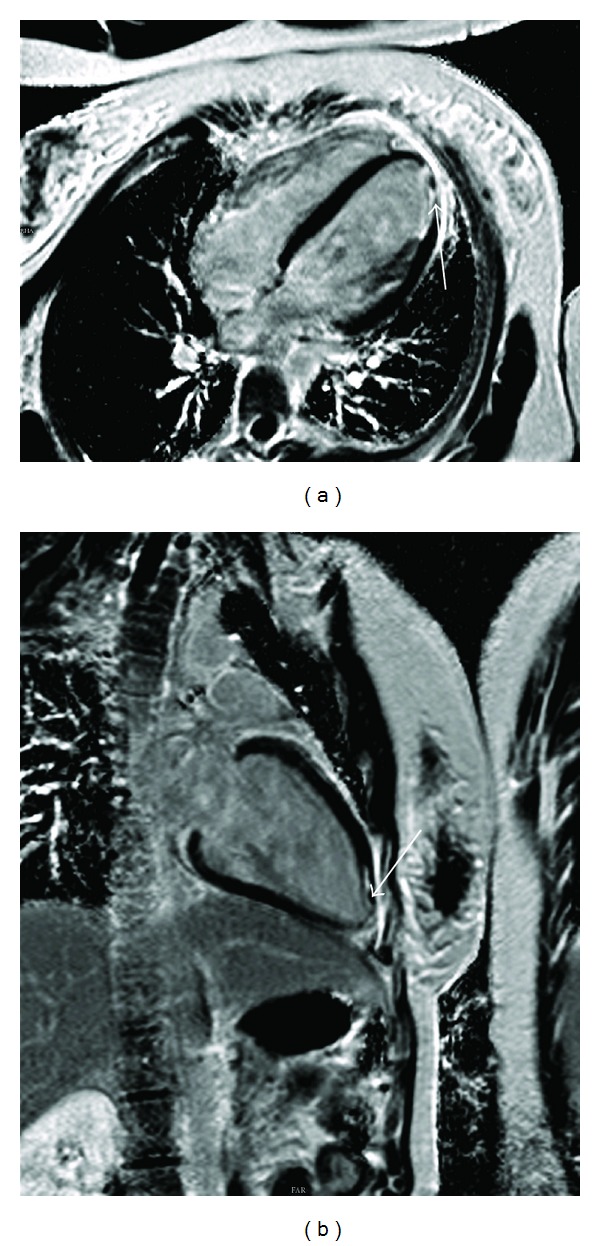
After ablation, a cardiac MRI study was performed. MRI images showed a slightly dilated left ventricle. End diastolic LV volume was 213 mL. End systolic LV volume was 112 mL. Stroke volume was 101 mL, LVEF was 47% and IVSDd was 11 mm. Gadolinium was used to evaluate myocardial perfusion. At the apicolateral side of the LV, a small area with transmural late enhancement was identified.

After ablation, a cardiac MRI study was performed. The quality of the postablation MRI images was excellent in contrast to preablation images. The MRI images (Figure 4) showed a dilated left ventricle. End diastolic LV volume was 213 mL. End systolic LV volume was 112 mL. Stroke volume was 101 mL. LVEF was 47%. IVSDd was 11 mm. Gadolinium was used to evaluate myocardial perfusion. At the apicolateral side of the LV, a small area with transmural late enhancement was identified. The successful ablation site was not in close proximity to the papillary muscle.

## 6. Discussion

In this patient with idiopathic incessant left ventricular ectopy and nonsustained VTs, it is unclear whether the underlying disease is a cardiomyopathy or a primary electrical disease. The arrhythmia was associated with a diminished LV function. The differential diagnosis was primary ventricular ectopy versus cardiomyopathy. It was not possible to determine whether the etiology was inflammatory, genetic, or metabolic in origin. Cardiac MRI showed a dilated left ventricle with decreased LV function (LVEF 40%). Cardiac biopsy was not performed. The 12 lead ECG showed a PVC with an RBBB morphology. It has been suggested that an RS interval of >100 msec in one or more precordial leads is suggestive for VT [5–8]. Berruezo et al. demonstrated that when the initial activation occurs in the epicardium, the intramyocardial delay of conduction produces a slurred initial part of the QRS complex (pseudo Δwave) [7]. Both the intrinsicoid deflection in V2, pseudodelta wave and the shortest RS complex had a longer duration in patients with VTs originating from the epicardium. Bazan et al. confirmed that these three parameters are useful in differentiating epicardial versus endocardial origin of left ventricular tachycardias [5, 6]. The morphology of ventricular ectopy in the present case meets the Brugada criteria for an epicardial origin. 

In general, these ventricular arrhythmias are difficult to treat with antiarrhythmic drugs. Lack of response to antiarrhythmic drug therapy is not unusual and does not necessarily indicate a diagnosis or prognosis. Endocardial and percutaneous epicardial ablation failed due to the localization of the arrhythmia focus in close proximity to the phrenic nerve. Intraoperative epicardial mapping and ablation was necessary to definitively localize and treat the source of the premature ventricular complexes.

## 7. Epicardial Ablation

The epicardial approach can be considered in patients with thrombus in the left ventricular cavity or in patients with metallic valve prostheses both in the aortic and mitral positions. It is not clear whether one should only use this approach after an endocardial failure or only when the ECG of the clinical ectopic activity/VT suggests an epicardial origin. In the present case, we used the combined endocardial and epicardial approach. It has been suggested that failure of endocardial ablation may be caused by substrates located intramurally or in the subepicardium, which are inaccessible to endocardial mapping and ablation techniques. As a matter of fact, the simultaneous approach may have several advantages such as lower cost and a better chance to map and ablate all inducible VTs. Some of the complications when performing endocardial VT ablation could occur with epicardial subxiphoid ablation, and in addition, the coronary arteries and the phrenic nerves should be avoided. The most important disadvantage of the epicardial technique is that a redo procedure will be hampered by pericardial adhesions, and for the same reason, epicardial ablation after cardiac surgery or after previous pericardial disease will generally not be possible. Experimental studies suggest that fatty tissue attenuates epicardial lesion formation, and this is one reason why epicardial ablation may fail. Therefore, an irrigated tip ablation catheter is preferred to produce more extensive and deep RF lesions. In the majority of patients, the epicardial subxiphoid approach is an alternative approach to deal with incessant ventricular ectopy and VT. Fan et al. delineated the course of the left phrenic nerve using pacing manoeuvres [2]. Buch et al. [3] described a novel method to prevent phrenic nerve capture during epicardial ablation by using a balloon catheter in the pericardial space to mechanically separate the left phrenic nerve from the ablation catheter. We did not use a balloon in this case. If percutaneous transpericardial RF energy delivery is associated with a high risk of collateral damage to important anatomical structures close to the origin of the ventricular arrhythmia, the balloon or a surgical approach should be considered. In the present case, the surgeon was able to ablate the VT focus avoiding damage to the phrenic nerve. At the latest followup, the patient had no ventricular ectopy postablation during Holter monitoring. LV function improved with a LVEF of 47%. 

## 8. Conclusion

This paper describes a patient with incessant left ventricular ectopy originating from the left ventricular epicardium close to the phrenic nerve. Delineation of the course of the phrenic nerve and recognition of phrenic nerve stimulation during RF energy delivery using an epicardial approach is critical to avoid damage to the phrenic nerve resulting in hemidiaphragmal paralysis. Surgical ablation is a safe option in selected patients. 

##  Conflict of Interests

The authors have no conflict of interests.

## Abbreviations

VT: Ventricular tachycardia
PVC: Premature ventricular complex
RBBB: Right bundle branch block
MRI: Magnetic resonance imaging
LVEF: Left ventricular ejection fraction
IVSDd: Interventricular septum diastolic diameter
RF: Radiofrequency
IV: Intravenous.
